# Genetic hybridization between C and R strains of *Spodoptera frugiperda* (Lepidoptera, Noctuidae) in Burkina Faso

**DOI:** 10.3897/BDJ.14.e212183

**Published:** 2026-11-03

**Authors:** Nongamanégré Kouanda, Grégoire Noël, Marcellin Cuma Cokola, Joachim Carpentier, Antoine Sanon, Athanase Badolo, Frédéric Francis

**Affiliations:** 1 Functional and Evolutionary Entomology, Gembloux Agro-Bio Tech, University of Liège, Belgium, Gembloux, Belgium Functional and Evolutionary Entomology, Gembloux Agro-Bio Tech, University of Liège, Belgium Gembloux Belgium; 2 Fundamental and Applied Entomology Laboratory, UFR SVT, University Joseph Ki-Zerbo, Ouagadougou, Burkina Faso, Ouagadougou, Burkina Faso Fundamental and Applied Entomology Laboratory, UFR SVT, University Joseph Ki-Zerbo, Ouagadougou, Burkina Faso Ouagadougou Burkina Faso; 3 Department of Crop Production, Faculty of Agriculture and Environmental Sciences, Université Evangélique en Afrique, Bukavu, Democratic Republic of Congo, Bukavu, Democratic Republic of the Congo Department of Crop Production, Faculty of Agriculture and Environmental Sciences, Université Evangélique en Afrique, Bukavu, Democratic Republic of Congo Bukavu Democratic Republic of the Congo

**Keywords:** *Spodoptera frugiperda*, corn, strain identification, heterozygotes, haplotypes

## Abstract

The fall armyworm (FAW), *Spodoptera frugiperda* (J.E. Smith), is a devastating pest that causes significant crop losses and threatens food security throughout sub-Saharan Africa. In Burkina Faso, FAW is present since 2017, but its diversity is poorly understood, which complicates control efforts. Morphological identification of the larvae collected in the field was followed by molecular characterization using the genetic markers cytochrome oxidase I (COI) and nuclear triose phosphate isomerase (Tpi). The larvae were collected from maize and sorghum fields in two of the country's agroecological zones. The analyses confirmed the coexistence of the two strains but identified a critical divergence. While the mitochondrial COI marker identified distinct maternal lineages corn strain (C) and rice strain (R), the nuclear Tpi gene revealed a high prevalence of heterozygotes. This provides evidence of frequent hybridization between populations in the field. Additionally, genetic analysis of populations based on both markers revealed high haplotype diversity and no evidence of significant genetic structure between agroecological zones. This indicates a panmictic population with high gene flow. These findings provide the first evidence of FAW CxR hybrids in Burkina Faso. This emphasises the significance of genetic diversity in the pest's capacity to adapt and underscores the necessity of management strategies that consider population genetics.

## Introduction

The fall armyworm (FAW), *Spodoptera frugiperda* (J.E. Smith) (Lepidoptera, Noctuidae), is native to the tropical and subtropical Americas (Luginbill 1928, Sparks 1979). It feeds on more than 350 plant species from 76 families, mainly Poaceae, Asteraceae and Fabaceae (Montezano et al. 2018). Since its first detection in West Africa in 2016 (Goergen et al. 2016), FAW has rapidly spread across the sub-Saharan Africa, the Maghreb, the Asian and European continents (Dahi et al. 2020, Prasanna et al. 2018, Cean et al. 2024). It is now listed among the European Union's 20 priority quarantine pests (UE 2019). In Burkina Faso, FAW became established in 2017 and is recognized as a major crop pest (MAAH 2018). Its high dispersal capacity, broad host range, insecticide resistance and ecological plasticity make it difficult to control (Palli et al. 2023). It has become a major pest of several economically important crops worldwide (Kenis et al. 2023). In Africa, initial loss estimates were $284 million and $198 million for Ghana and Zambia respectively, and between $2.5 and $6.3 billion across 12 African countries in 2017 (Day et al. 2017). In Burkina Faso, the estimated yield loss caused by FAW is between 17.6 and 26.2% per hectare (FAO 2023, Yaméogo et al. 2024).

In its native range, FAW comprises two host-associated strains: the corn strain (C) and the rice strain (R) (Nagoshi et al. 2007b, Nagoshi et al. 2012). Although morphologically indistinguishable and capable of hybridization (Nagoshi et al. 2006), they differ in host preference, with the C strain primarily associated with maize and sorghum, and the R strain with rice and other small grasses (Pashley 1986). Molecular markers, particularly the mitochondrial COI gene and the nuclear Tpi gene, are widely used for strain identification and hybrid detection (Nagoshi et al. 2006b, Nagoshi 2010). However, in Africa, COI haplotypes frequently indicate the R strain in populations collected on maize (Assefa 2019), reflecting extensive hybridization and discordance between mitochondrial and nuclear markers rather than true host-associated strain identity (Kuate et al. 2019, Acharya et al. 2021, Nagoshi 2025). In Africa, both strains have been identified in many countries (Cock et al. 2017, Sarr et al. 2021). Importantly, several studies have documented the co-occurrence of both mitotypes on the same host plant in West and Central Africa. For instance, (Kuate et al. 2019) demonstrated the presence of COI-RS haplotypes on maize and sorghum in Cameroon. In contrast, (Nagoshi et al. 2021) reported that the C strain is dominant in Africa, with the R strain being rare or absent. These findings suggest that, unlike in parts of the Americas, the two strains are not ecologically segregated in Africa, but instead hybridise freely, resulting in a panmictic population structure with mixed genetic backgrounds (Nagoshi et al. 2021). This apparent paradox, whereby R-strain mitochondrial markers are found on C-strain host plants, is now interpreted as evidence of widespread hybridization and genetic introgression, rather than as true R-strain ecology (Nagoshi 2025).

Although FAW has been found on numerous plants in Burkina Faso (Compaoré et al. 2023), research on the pest's strains in that country is notably scarce (Sarr et al. 2021). However, detecting FAW strains in a given geographic area not only helps understand the geographic distribution of strains and their dispersal patterns (Tessnow et al. 2025), but also provides information on high-risk crops (Nagoshi 2025), and contributes towards shaping pest control strategies and policies. Furthermore, understanding whether the R-strain mitochondrial signatures observed on maize correspond to true R-strain individuals (with R nuclear background) or to hybrids is critical for accurate pest management and host plant risk assessment. Such results will contribute to improving monitoring methods using pheromone traps (Meagher et al. 2013), and optimizing control strategies. This study aims to identify the strains of FAW present in Burkina Faso and to study their genetic variability across two agro-climatic zones, with particular attention to the discordance between COI and Tpi markers as an indicator of ongoing hybridization.

## Material and methods

### Specimen collection

FAW larvae were collected from 23 localities across two agro-climatic zones (Fig. 1) in Burkina Faso between February and September 2023 (Suppl. material 1), during the dry and rainy seasons. Sampling was conducted in maize, sorghum, millet, rice, vegetable fields, and pasture. In each field, larvae were collected from five randomly selected points following a W-shaped sampling pattern (Prasanna et al. 2018). Larvae were transported to the laboratory in aerated containers and identified individually based on diagnostic morphological characteristics, such as the inverted Y-shaped mark on the head capsule and the four-dot pattern on the eighth abdominal segment (OPPE 2015). Confirmed larvae were preserved individually in 96% ethanol at -20°C until DNA extraction.

### DNA extraction

DNA was extracted from 24 FAW larvae. Each larva was washed three times with sterile distilled water before DNA extraction. A small thoracic segment (approx. 20 mg) was excised using a sterile scalpel and placed in a 1.5 mL microtube. DNA was extracted using the QIAGEN Kit, DNeasy Blood and Tissue Kit (QIAGEN GmbH, Qiagen Strass 1 Germany) according to the manufacturer's instructions. DNA concentration and purity were assessed using a Nanodrop spectrophotometer. A final volume of 100 µL per DNA sample was obtained and stored at -20° C until PCR amplification.

### Polymerase Chain Reaction (PCR) amplification and DNA sequencing

The concentration of all DNA samples was diluted to 10 ng/µl. A 50 µl PCR mix was prepared containing 25 µl of Q5®High-Fidelity Master Mix, 2.5 µl of each primer, 10 µl of molecular grade water, and 10 µl of DNA template. The PCR protocol began with an initial denaturation at 98°C for 30 seconds, followed by 35 cycles of denaturation at 98°C for 30 seconds, annealing at 56°C for 30 seconds, and extension at 72°C for 30 seconds, with a final extension at 72°C for 2 minutes. A fragment of the mitochondrial COI gene was amplified using primers 891F (5'-TACACGAGCATATTTTACATC-3') and 1472R (5'-GCTGGTGGTAAATTTTGATATC-3') to produce 603 bp fragment. The Tpi exon segment was amplified using primers 412F (5'-CCGGACTGAAGGTTATCGCTTG-3') and 1140R (5'-GCGGAAGCATTCGCTGACAACC-3') to produce a variable length fragment due to insertion and deletion mutations (Nagoshi et al. 2019). For fragment isolation, 5µL of 6X DNA Loading Dye Buffer was added to each amplicon. Samples were electrophoresed on a 1% agarose gel at 37° C for 2 hours and visualized under UV light. DNA was purified using a gel and PCR clean-up kit according to the manufacturer's protocol and sent for sequencing to Eurofins Genomics Europe Pharma and Diagnostics Products (Germany).

### Sequences analyses and fall armyworm strains identification

The sequences obtained from Eurofins Genomics were individually checked against the GENBANK database using BLASTN (https://blast.ncbi.nlm.nih.gov/Blast.cgi). Sequence analysis was performed following the standardized nomenclature and criteria established by Nagoshi et al. (2017) and Nagoshi et al. (2019). All sequences were assembled and aligned using Clustal W from MEGA 11, version 11.0.13 (Thompson et al. 1997) after quality control using BioEdit 7.7.1 (Alzohairy 2011). This alignment was used to detect nucleotide polymorphisms for identifying R and C strains. The COI gene segment was used for initial strain identification. The single nucleotide polymorphism (SNP) at site mCOI_1164_ D (numbering from the predicted translational start site) was used as the primary diagnostic marker, where T₁₁₆₄ identifies the rice-strain (COI-RS) and A₁₁₆₄ or G₁₁₆₄ identify the corn-strain (COI-CS). A phylogenetic tree of COI sequences was reconstructed using the neighbor-joining method in MEGA 11 software verion 11.0.13 (Tamura et al. 2021). The reliability of tree topology was evaluated by 1.000 bootstrap replicates. Sequences from strain R (NT-RS1, Africa RSa4, Africa RSa3) and strain C (Africa CSa1, NT-CS1) from Genbank (Nagoshi et al. 2020, Piggott et al. 2021) were used. The nuclear Z-chromosome linked Triose-Phosphate Isomerase (Tpi) gene, specifically the fourth exon segment (Tpi E4), provided complementary strain identification. The SNP at site g Tpi 183Y (numbering from the 5' start of exon 4) served as the diagnostic marker, where C₁₈₃ indicates the corn-strain (Tpi-C) and T₁₈₃ indicates the rice-strain (Tpi-R) (Nagoshi et al. 2019). This analysis was performed using R 4.5.2 (R Core Team 2025) based on the ab1 files, followed by a manual analysis of the electropherograms for each sequence.

### Haplotype characterization

Specimens were classified into COI haplotypes based on polymorphisms at the strain-diagnostic site mCOI_1164_D and six additional strain-biased sites (mCOI1125Y, mCOI1176Y, mCOI_1182_Y, mCOI_1197_R, mCOI_1216_W, and mCOI_1287_R). For the Tpi gene, male specimens (ZZ chromosomes) may be heterozygous (Tpi-H). The genotypes of heterozygotes were determined as TpiC-YY=TpiCa1/TpiCa2, TpiH-CC=TpiRa1/TpiCa1, and TpiH-YY=TpiRa1/TpiCa2. Haplotype frequencies were calculated according to the inheritance patterns of the mitochondrial COI and nuclear Tpi genes. Each specimen contributed one maternally inherited COI haplotype. For the Z-linked Tpi gene, haplotype frequencies were adjusted following (Nagoshi et al. 2019), assuming a 1:1 sex ratio (1.5 Tpi copies per specimen). Adjusted counts were obtained by summing 1.5 times the number of homozygotes for each haplotype plus the relevant heterozygotes carrying that allele. A Mantel test assessed the relationship between genetic and geographic distances. Genetic differentiation and gene flow were estimated using DnaSP v.6.12.03 (Rozas et al. 2017). Differences in haplotype frequencies between the two agroecological zones were tested using Fisher's exact test, with p-values estimated by Monte Carlo simulation (100,000 replications). Haplotype networks were generated with PopArt v.1.7 (Snell et al. 2002).

## Results

### FAW identification and strain-specific haplotype characterization

All larvae were morphologically identified as *S. frugiperda*. They were only found in maize and sorghum plots. The mitochondrial (COI) and the nuclear (TPI) markers confirmed this identity, with all sequences closely matching references of FAW sequences in GenBank. Analysis of the strain-diagnostic single nucleotide polymorphisms (SNPs) revealed the complex population structure of FAW in Burkina Faso. COI analysis identified the corn-strain (C) marker (A/G at position 1164) in 66.7% (16/24) of sequenced individuals and the rice-strain (R) marker (T at position 1164) in 33.3% (8/24). All genes have been deposited in GenBank with the following accession numbers: PX795207-PX795226, PZ130026, PZ130027, PZ139264 and PZ139265.

However, Tpi gene revealed a different picture. Using the gTpi183Y diagnostic site, the vast majority of specimens (75%, or 18/24) were found to be heterozygous (TpiH), displaying both C and T nucleotides at this position. Only 8.3% (2/24) were homozygous for the corn strain allele (TpiC), while 16.7% (4/24) were homozygous for the rice strain allele (TpiR) (Table 1). All TPI genes have also been deposited in GenBank, with the accession numbers listed in Table 1. The combined COI and *Tpi* data revealed a high degree of marker discordance. The most common configuration was COI-RS TpiH, found in 62.5% of specimens, representing individuals with a rice-strain mitochondrial background but a heterozygous nuclear genotype. The configuration COI-CS TpiH was also frequent (16.7%). In contrast, the concordant configurations typical of Western Hemisphere populations (COI-CS TpiC and COI-RS TpiR) were rare, representing only 4.2% and 8.3% of our samples, respectively. The remaining 8.3% were COI-RS TpiC. This widespread discordance, particularly the high frequency of Tpi heterozygotes, provides strong evidence of hybridization and inter-strain mating within Burkina Faso populations of FAW.

### Geographical distribution and population structure of fall armyworm in Burkina Faso

The Mantel test revealed no significant correlation between genetic and geographical distances (p-value = 0.872; r = -0.114) among the sampled individuals (Suppl. material 2). Thirteen and eighteen haplotypes were detected among the sequences analyzed for the COI and TPI genes, respectively, indicating a high level of haplotype diversity (Table 2). However, no significant differences in haplotype distribution were detected by Ficher’s exact tests for either the COI gene (p-value = 0.07763) or the TPI gene (p-value = 0.8286) between the two zones. Estimates of gene flow for both genes revealed significant migration between populations in the two agroclimatic zones.

### Haplotype networks and evolutionary relationships

The minimum spanning network analysis identified 13 haplotypes for the COI gene and 18 for the Tpi gene. The COI network showed a clear separation between the corn-strain (C) and rice-strain (R) haplotypes (Fig. 2a). The Tpi network was more complex, with a star-like pattern centred on common haplotypes and numerous derived haplotypes, reflecting the high degree of heterozygosity and sequence variation (Fig. 2b). Certain haplotypes (hap_1, 2, and 3) for the TPI gene and hap_4 for the COI gene were found in both agroclimatic zones.

The neighbor-joining phylogenetic tree of COI sequences (Fig. 3) clearly grouped the specimens alongside the reference strains, thus confirming the identity of the C and R haplotypes presents in Burkina Faso. The sequences of FAW strains confirmed as R strain showed close relationships with haplotypes previously identified in other parts of Africa (MN551263, MN551265 and MN551266) and Australia (MW454868).

## Discussion

Our results confirmed the presence of both corn (C) and rice (R) strains in Burkina Faso, and reveal extensive strain-marker discordance and a high frequency of Tpi heterozygotes. The COI and Tpi data showed substantial incongruence, with the dominant configuration across our samples being COI-RS Tpi, followed by COI-CS TpiH. Concordant strain signatures (COI-CS TpiC or COI-RS TpiR) were rare. This strong discordance is consistent with the now well-established pattern across Africa and Asia, where the COI-RS, TpiC and hybrid configurations dominate (Nagoshi et al. 2019, Nagoshi et al. 2021). In the Western Hemisphere, COI and Tpi markers agree in ~80% of individuals and Tpi heterozygotes were uncommon, reflecting limited inter-strain mating (Nagoshi et al. 2021, Tessnow et al. 2025). Conversely, studies of African populations have revealed widespread interbreeding, which has often been taken as proof of hybridisation between strains following the FAW’s introduction to Africa. The presence of a small dispersal unit containing both strains would result in high rates of hybridisation, due to assortative mating being weakened and hybrids having selective advantages. These phenomena are commonly observed during pest invasions (Nagoshi et al. 2019, Wang et al. 2025). Our results, showing 75% heterozygotes for the Tpi gene in Burkina Faso, support this hypothesis.

One of the main findings was the high frequency of COI-RS (rice strain mitochondrial marker) and Tpi heterozygotes in larvae collected mainly from maize and sorghum, the preferred hosts of the C strain. This apparent contradiction has been explained in previous African studies. Nagoshi (2025) showed that the mitochondrial COI marker may incorrectly identify R-strain individuals on C-strain host plants because historical hybridization has uncoupled mitochondrial and nuclear genomes. Similarly, Kuate et al. (2019) and Sarr et al. (2021) reported the coexistence of both mitotypes (COI-CS and COI-RS) on maize, indicating that the two strains are not ecologically separated in Africa. Sisay et al. (2024) further demonstrated that inter-strain mating occurs readily and produces viable offspring. Thus, the presence of COI-RS on maize and sorghum reflects genetic introgression rather than true R-strain host preference. Low mitochondrial and nuclear haplotype diversity, consistent with previous African studies (Nagoshi et al. 2019, Acharya et al. 2021), supports a genetic bottleneck during invasion. The widespread COI-CSa1 haplotype reinforces the proposed Florida-Caribbean origin of African populations. Phylogenetic analyses also grouped both C- and R-strain haplotypes with African and Australian references, supporting a single or limited introduction followed by rapid spread.

The analyses revealed no significant genetic differences between FAW individuals from the two zones of Burkina Faso. Genetic analyses and the Mantel test detected no spatial genetic structure, while the observed haplotypes differed by only a few mutations (Hudson et al. 1992). Because FAW was first reported in Africa in 2016 (Goergen et al. 2016) and in Burkina Faso in 2017, these findings support a recent invasion followed by rapid population expansion, consistent with previous studies (Nagoshi et al. 2007a, Nagoshi et al. 2019, Sisay et al. 2024). FAW's high dispersal capacity (Johnson 1987), combined with continuous host availability throughout the year (Montezano et al. 2018), likely maintains strong gene flow and connectivity among populations (Nei 1982). Similar temperatures across both climatic zones, remaining above the developmental threshold (≈10°C), further promote continuous reproduction and genetic homogenization. These results support implementing wide-area rather than region-specific management strategies.

Whether the R strain occurs in Africa remains a key question in FAW research. Although the rarity of TpiR initially suggested that the R strain was absent or declining in the Eastern Hemisphere (Nagoshi et al. 2021), recent studies have confirmed its presence and additional introductions from West Africa (Nagoshi 2025). In our study, COI-RS was common, whereas TpiR remained rare (≈16% of haplotypes). This discordance reflects the decoupling of COI from strain identity, making mitochondrial markers unreliable predictors of host preference (Tessnow et al. 2022). The high frequency of Tpi heterozygotes indicates extensive inter-strain admixture (Wang et al. 2025), with persistent COI-RS haplotypes reflecting historical hybridization rather than R-strain host association (Acharya et al. 2021, Sisay et al. 2024, Nagoshi 2025).

The extensive hybridization observed in Burkina Faso has important implications for FAW management. First, host-use predictions become unreliable when mitochondrial markers no longer reflect strain identity, making COI-based diagnostics insufficient for crop risk assessment. Second, although the COI-RS haplotype predominates in maize and sorghum, this does not imply a greater risk to rice. Instead, it reflects an admixed population capable of exploiting multiple hosts regardless of mitochondrial background (Nagoshi et al. 2021). Third, hybridization may accelerate insecticide resistance by promoting the combination of resistance alleles or novel detoxification traits. Fourth, biological control strategies should be validated locally, as hybrid populations may differ from the canonical American C- and R-strains in traits affecting pheromone response or natural enemy performance. Finally, the persistence of COI-RS despite the rarity of TpiR highlights the need to monitor potential host-range expansion, particularly in rice and pasture ecosystems.

These findings emphasize the need for broader research on FAW population dynamics in Burkina Faso, including: (i) expanded sampling of host plants, especially rice and pasture, to determine whether true TpiR individuals persist or whether COI-RS reflects historical hybridization; (ii) cross-breeding experiments to compare hybrid and parental fitness under local conditions; (iii) landscape-scale studies integrating climate, cropping systems and agronomic practices to model dispersal; and (iv) genome-wide analyses to resolve population structure and identify selective pressures in admixed populations.

## Conclusions

This study provides the first molecular characterization of FAW populations in Burkina Faso. It reveals low genetic diversity and no geographic structure, which is consistent with a recent introduction followed by extensive dispersal. Discordance between COI and Tpi markers, alongside the high frequency of Tpi heterozygotes, suggests widespread interstrain hybridisation. The presence of R-strain mitochondrial markers on maize and sorghum likely reflects genetic admixture rather than host-associated ecology. Meanwhile, the predominance of a C-strain nuclear background highlights the limitations of COI for strain identification and host-use prediction. These findings emphasize the importance of ongoing molecular surveillance using nuclear markers and the need for integrated pest management strategies adapted to admixed FAW populations.

## Supplementary Material

A841E419-4C1C-5A44-B22C-02233E9D05FC 10.3897/BDJ.14.e212183.suppl1 Supplementary material 1 Number of larvae collected by location Data type Table Brief description Number of larvae collected by location. The collection site is identified by the name of the village. Larvae were found on corn and sorghum. File: oo_1742011.docx https://binary.pensoft.net/file/1742011 Nongamanégré KOUANDA

BF833FAF-FF20-56D0-88ED-27361ED6AD0A 10.3897/BDJ.14.e212183.suppl2 Supplementary material 2 Relationship between genetic distance and geographic distribution Data type Image Brief description Relationship between genetic distance and geographic distribution in fall armyworm populations. Blue dots show pairwise comparisons between individuals. The red line indicates the fitted linear regression, and the grey band shows its 95% confidence interval. File: oo_1725283.jpg https://binary.pensoft.net/file/1725283 Grégoire Noël

## Figures and Tables

**Figure 1.**
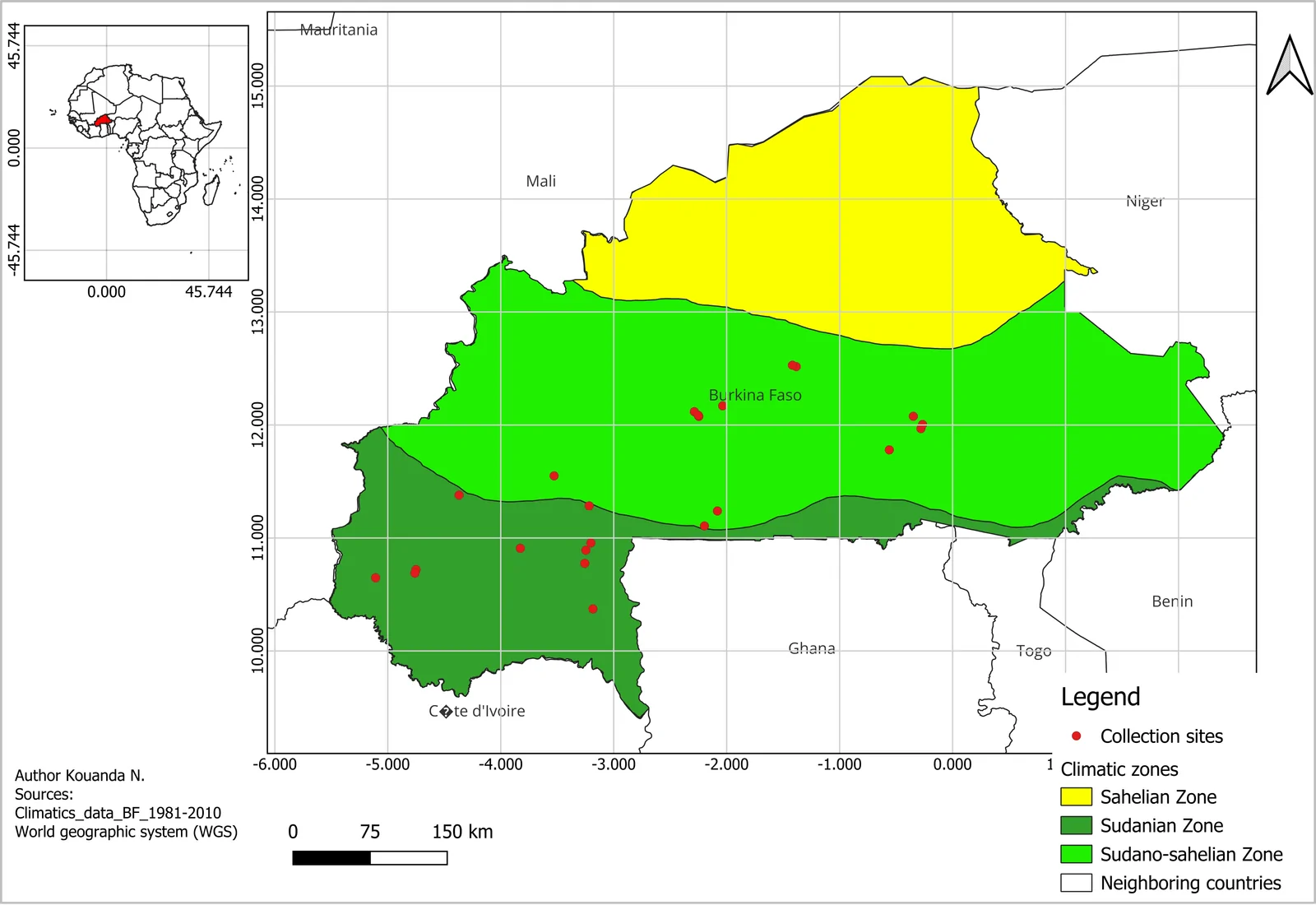
Map of Burkina Faso showing the distribution of FAW larval collection sites. Samples were collected from 23 localities (red dots) across the Sudanian (dark green) and Sudano-Sahelian (green) agroclimatic zones. Only the plots where larvae were collected are shown in the graph. The Sahelian zone (yellow) was not sampled.

**Figure 2.**
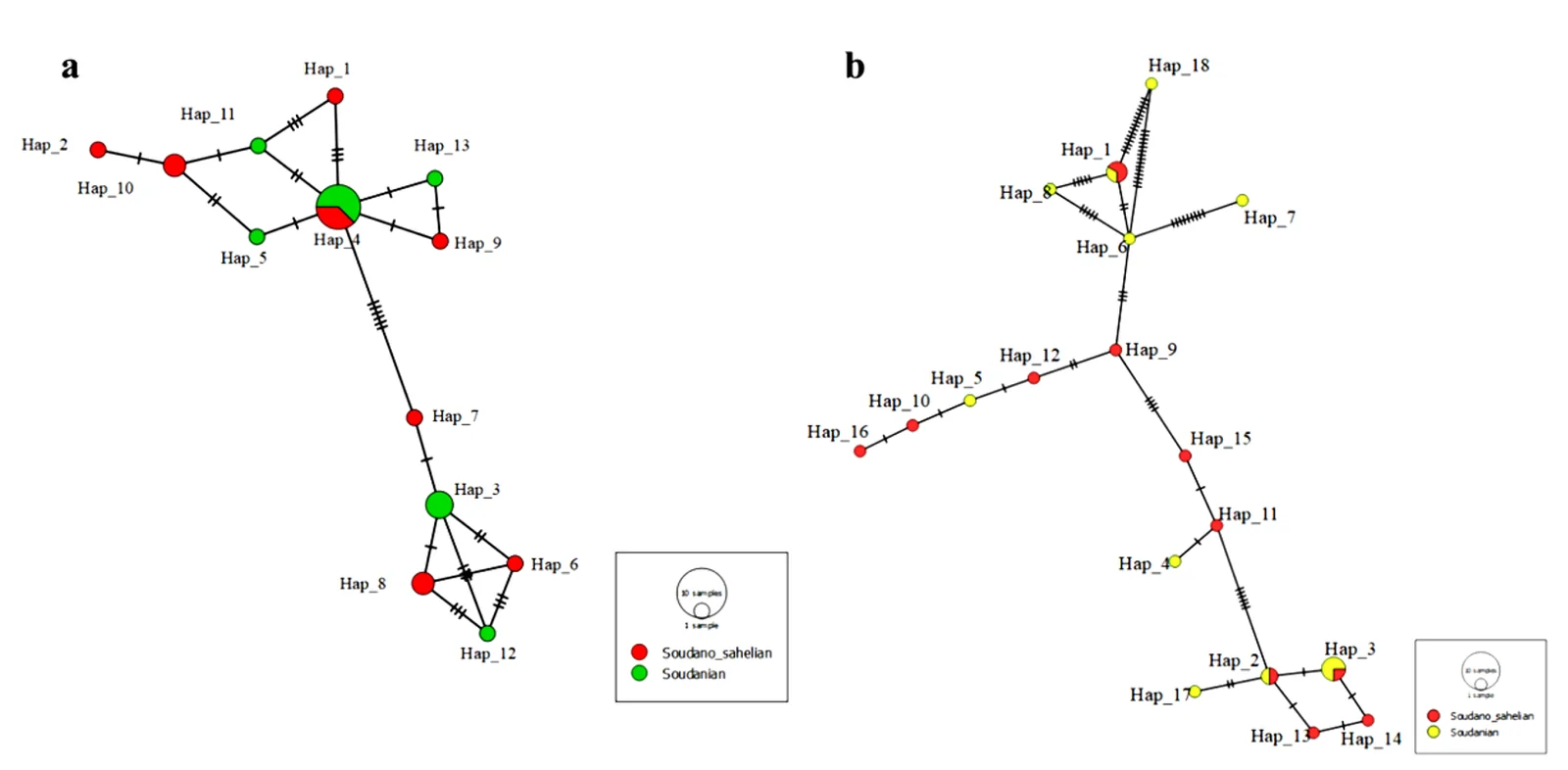
Haplotype networks of fall armyworm in Burkina Faso based on mitochondrial COI (a) and nuclear Tpi sequences (b). Circles represent unique haplotypes, with size proportional to frequency. Lines indicate single mutational steps, and colors denote the agroclimatic zone of each haplotype.

**Figure 3.**
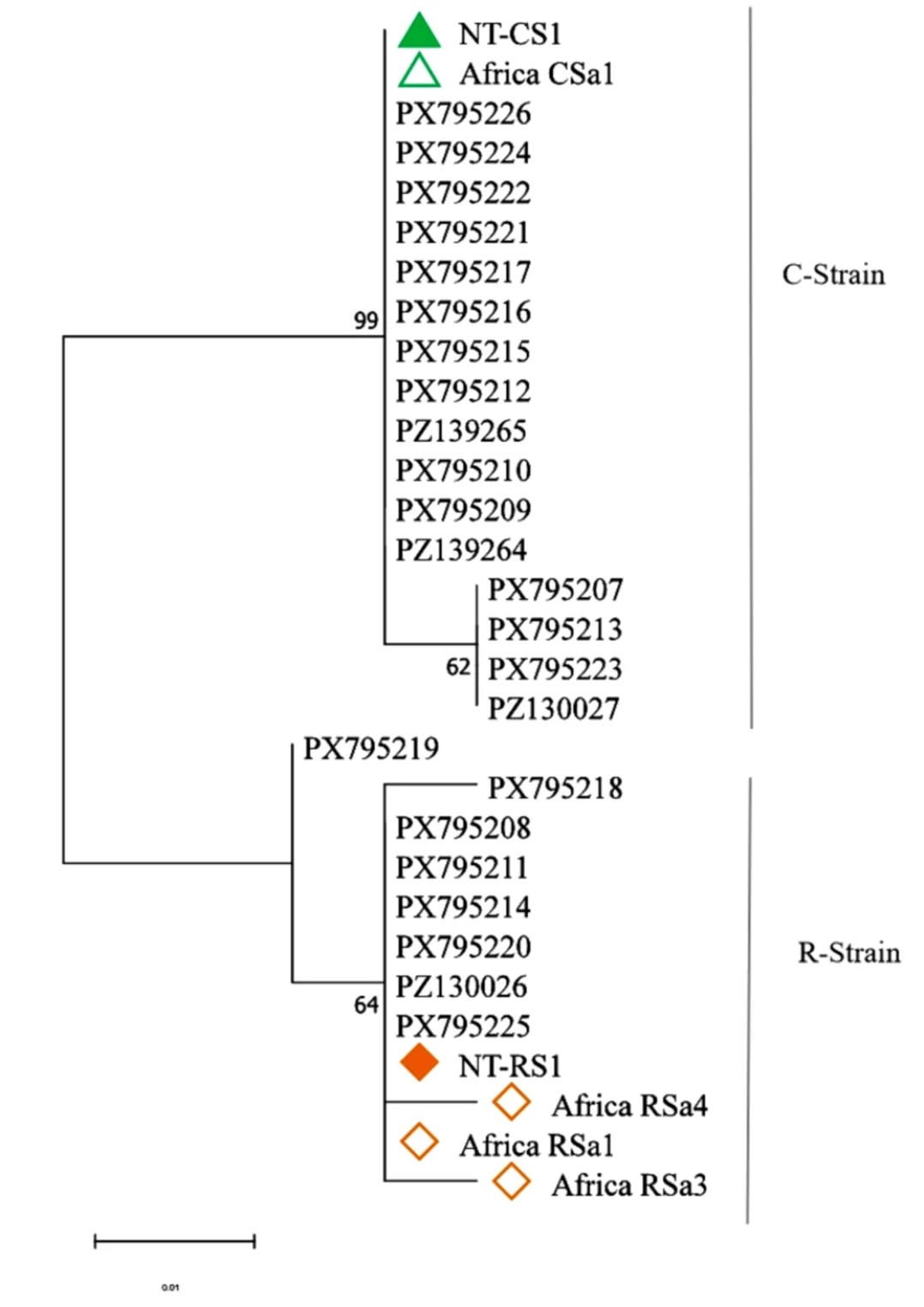
Phylogenetic tree of fall armyworm COI haplotypes from Burkina Faso. The neighbor-joining tree shows the relationships between sequences from this study (accession numbers) and reference corn (C) and rice (R) strain sequences from Africa and Australia. Bootstrap values indicate node support. Green symbols represent the African (triangle; Africa CSa1) and Australian (filled triangle; NT-CS1) C strains, while orange symbols represent the African (Africa RSa1,3,4) and Australian (NT RSa4) R strains.

**Table 1.** Distribution of fall armyworm strains according to climatic zone based on Tpi marker.

**Agroecological zone**	**Host plant**	**TPI Accession number**	**gTpi 183Y**	**Strain**
Sudanian	Maize	PZ161590	T	R Strain
Sudanian	Maize	PZ161591	C	C Strain
Sudanian	Maize	PZ161592	A	Heterozygote
Sudanian	Maize	PZ161572	A	Heterozygote
Sudanian	Maize	PZ161573	A	Heterozygote
Sudanian	Maize	PZ161574	C	C Strain
Sudanian	Maize	PZ161575	A	Heterozygote
Sudanian	Maize	PZ161576	A	Heterozygote
Sudanian	Maize	PZ161577	T	R Strain
Sudanian	Maize	PZ161578	T	R Strain
Sudanian	Sorghum	PZ167435	A	Heterozygote
Sudanian	Sorghum	PZ161579	G	Heterozygote
Sudano-sahelian	Maize	PZ161570	A	Heterozygote
Sudano-sahelian	Maize	PZ161571	T	R Strain
Sudano-sahelian	Maize	PZ161580	A	Heterozygote
Sudano-sahelian	Maize	PZ161581	A	Heterozygote
Sudano-sahelian	Maize	PZ161582	A	Heterozygote
Sudano-sahelian	Maize	PZ161583	A	Heterozygote
Sudano-sahelian	Maize	PZ161584	A	Heterozygote
Sudano-sahelian	Maize	PZ161585	A	Heterozygote
Sudano-sahelian	Maize	PZ161586	G	Heterozygote
Sudano-sahelian	Maize	PZ161587	A	Heterozygote
Sudano-sahelian	Maize	PZ161588	A	Heterozygote
Sudano-sahelian	Maize	PZ161589	A	Heterozygote

**Table 2.** Genetic variability analysis of Tpi and COI gene of fall armyworm in Burkina Faso.

**Genes**	**Regions**	**Numbers of sequence**	**Number of segration sites (S)**	**Numbers of haplotypes (h)**	**Nucleotide diversity (π)**
COI	Sudano-Sahelian	12	15	8	0,019
	Sudanian	12	11	6	0,014
Tpi	Sudano-Sahelian	12	16	11	0,029
	Sudanian	12	37	10	0,048
