# Supplementary material for: Genetic hybridization between C and R strains of Spodoptera frugiperda (Lepidoptera, Noctuidae) in Burkina Faso
Source: Biodivers Data J. 2026 Nov 3;14:e212183. doi: 10.3897/BDJ.14.e212183 (PMC13752709; doi:10.3897/BDJ.14.e212183)
Supplement: Supplementary file 1 [file bdj-14-e212183-s001.docx]

**Supplementary material T1:** Number of larvae collected during this study, by location. The collection site is identified by the name of the village. Larvae were found on corn and sorghum.

| **Collection site** | **Host plant** | **Lat.** | **Long.** | **Number of larvae** |
| --- | --- | --- | --- | --- |
| Tanghin-Woobdo | Maize | 12,1694 | -2,03555 | 40 |
| Sourgou | Maize | 12,1177 | -2,2862 | 9 |
| Bérégadougou | Maize | 10,7201 | -4,7504 | 22 |
| Nafona 2 | Maize | 10,6891 | -4,7603 | 29 |
| Sindou | Maize | 10,6473 | -5,1079 | 30 |
| Badara | Maize | 11,3793 | -4,3694 | 67 |
| Dikando | Maize | 10,3722 | -3,1849 | 21 |
| Bapla | Maize | 10,8914 | -3,2467 | 19 |
| Tienkoura | Maize | 10,7750 | -3,2561 | 8 |
| Koper | Sorghum | 10,9559 | -3,2023 | 14 |
| Karaba | Maize | 11,2842 | -3,2179 | 17 |
| Poumpoi | Maize | 11,5500 | -3,5279 | 31 |
| Tansoabtenga | Maize | 11,9678 | -0,2801 | 6 |
| Ouédgo | Maize | 12,0770 | -0,3477 | 12 |
| Pagou | Maize | 11,7795 | -0,5600 | 12 |
| Tansoabtenga | Maize | 12,0044 | -0,2657 | 8 |
| Loumbila | Maize | 12,5166 | -1,3841 | 30 |
| Noungou | Maize | 12,5290 | -1,4181 | 51 |
| Sabou | Maize | 12,0758 | -2,2465 | 30 |
| Sabou | Maize | 12,0867 | -2,2529 | 28 |
| Karangaso | Maize | 10,9085 | -3,8264 | 30 |
| Kayéro | Maize | 11,2386 | -2,0820 | 28 |
| Dabiou | Maize | 11,1065 | -2,1969 | 7 |
